# CYP2D6 testing to predict response to tamoxifen in women with breast cancer: Pharmacogenomic

**DOI:** 10.1371/currents.RRN1176

**Published:** 2010-10-01

**Authors:** Issa Dahabreh, Teruhiko Terasawa, Peter Castaldi, Thomas A Trikalinos

**Affiliations:** ^*^ICRHPS, Tufts Medical Center; ^†^Department of Internal Medicine, Fujita Health University School of Medicine; ^‡^Tufts Medical Center and ^§^Tufts Evidence-based Practice Center, Institute for Clinical Research and Health Policy Studies, Tufts Medical Center

## Abstract

Tamoxifen, a selective estrogen receptor modulator, is the standard of care for premenopausal women with estrogen or progesterone receptor-positive breast cancer and a valid option for treating post-menopausal women. However, a substantial number of tamoxifen-treated patients relapse following surgical resection, while others remain disease-free for many years. It appears that the primary effectors of tamoxifen activity are its active metabolites, rather than tamoxifen itself. Cytochrome P450 (CYP) enzymes, CYP2D6 in particular, play a major role in the metabolism of tamoxifen to active metabolites. More than 75 germline CYP2D6 variants have been identified.

A test predicting lack of response to tamoxifen could supplement information used by clinicians and patients in treatment decision-making. For example, physicians and patients may opt to switch to an alternative therapy upfront.

## **Clinical Scenario**

Testing of women with non-metastatic breast cancer to predict those who will not respond to tamoxifen therapy could inform decisions regarding choice of alternative treatment strategies including chemotherapy or the use of aromatase inhibitors (for post-menopausal women in particular [1]. 

##  **Test Description**

- Analysis of multiple single nucleotide polymorphisms, deletions or duplications in *CYP2D6* by DNA-based methods.
- Genotype-based prediction of CYP2D6 enzymatic activity ( [2] [3] [4]; see also Links section) categorizes patients into:
  - Ultra metabolizers (carrying multiple or duplicated functional alleles)
  - Extensive metabolizers (carrying “normal” function alleles)
  - Intermediate metabolizers
  - Slow metabolizers (carrying only no-or low-function alleles)  

## **Public Health Importance**

- It is estimated that approximately 192,000 U.S. women will be diagnosed with breast cancer and that 40,170 women will die of the disease [5].  More than 80 percent of all breast cancers express estrogen or progesterone receptors, and are candidates for endocrine therapy, including tamoxifen treatment. 
- An individual-patient data meta-analysis of 194 randomized controlled trials (145,000 patients) has demonstrated that tamoxifen reduces the risk of breast cancer relapse by about 50 percent and the risk of breast-cancer specific mortality by about 30 percent [1]. 
- There are effective treatment strategies that do not include tamoxifen.

## **Published Reviews, Recommendations and Guidelines**

**Systematic evidence reviews**

Agency for Healthcare Research and Quality (AHRQ), Evidence Report/Technology Assessment [6]. 

**Recommendations by independent group**

There are no recommendations by an independent group.

**Guidelines by professional groups**

American Society of Clinical Oncology clinical practice guideline update on the use of pharmacologic interventions including tamoxifen, raloxifene, and aromatase inhibition for breast cancer risk reduction: “Given the limited evidence, *CYP2D6* testing is currently not recommended in the preventive setting” [7]. 

## **Evidence Overview**

***Analytic Validity*: **Test accuracy and reliability in defining *CYP2D6* genotypes

Based on an AHRQ Evidence Report on antidepressants (includes reference to 2 FDA documents on the Roche Amplichip®) [8]:

- Very limited published data on few *CYP2D6* polymorphisms, and with important methodological shortcomings.
- Reported sensitivity and specificity were between 94 and 100 percent, but the confidence intervals were wide because of limited sample sizes.

- High analytic validity may be expected for testing of common *CYP2D6* alleles given that the utilized methods (mainly Taqman assays) are fairly standardized.
- Frequency of failed tests is unclear.

***Clinical Validity*: **Test accuracy and reliability in predicting clinical outcomes such as progression-free or overall survival.

- Based on an AHRQ Draft Technology Assessment that included 13 studies [6].
  - Large between-study variability in classifying genotypes to extensive, intermediate or slow metabolizers.
  - Most studies evaluated surrogate endpoints, such as disease- or recurrence free survival. Results were inconsistent in direction and formal statistical significance.
  - A few evaluated overall survival. None demonstrated any significant differences in overall survival by *CYP2D6* status.
  - Most reviewed studies had methodological shortcomings.
- Based on an earlier-published systematic review of 10 studies (included in the above review): Study results on the association between CYP2D6 status and breast cancer recurrence are “widely heterogeneous with relative-risk estimates outside the range of reasonable bounds”[3].
- Recent additions to the literature include:
  - A cohort of 1325 post-menopausal women with breast cancer who received tamoxifen following surgery demonstrated that carrying two functional *CYP2D6* alleles is associated with significantly improved event- and disease-free survival, but did not find significant associations with overall survival [9]. This study partially overlaps with studies included in the aforementioned reviews [10].
  - A study of 282 women receiving tamoxifen monotherapy demonstrated that recurrence-free survival increases with the number of functional *CYP2D6* alleles [11].

***Clinical Utility*: **Net benefit of test in improving health outcomes.

- No clinical trial has evaluated the net benefit of testing versus no testing in improving health outcomes
- We did not identify any modeling analysis that compared the expected benefits and harms of patient management strategies that are informed by *CYP2D6 *testing versus patient management strategies that are not informed by such testing.

## Links

Human Cytochrome P450 (CYP) Allele Nomenclature Committee, CYP2D6 allele nomenclature (http://www.cypalleles.ki.se/cyp2d6.htm, last accessed: March 15th, 2010). 

Last updated: *March 15, 2010*
